# Fabrication of bifunctional core-shell Fe_3_O_4_ particles coated with ultrathin phosphor layer

**DOI:** 10.1186/1556-276X-8-357

**Published:** 2013-08-21

**Authors:** Timur Sh Atabaev, Hyung-Kook Kim, Yoon-Hwae Hwang

**Affiliations:** 1 Department of Physics and Astronomy, Seoul National University, Seoul 151-747, Republic of Korea; 2 Department of Nanomaterials Engineering, Pusan National University, Miryang 627-706, Republic of Korea

**Keywords:** Nanocomposites, Bifunctional composite particles, Superparamagnetic behavior

## Abstract

Bifunctional monodispersed Fe_3_O_4_ particles coated with an ultrathin Y_2_O_3_:Tb^3+^ shell layer were fabricated using a facile urea-based homogeneous precipitation method. The obtained composite particles were characterized by powder X-ray diffraction, transmission electron microscopy (TEM), quantum design vibrating sample magnetometry, and photoluminescence (PL) spectroscopy. TEM revealed uniform spherical core-shell-structured composites ranging in size from 306 to 330 nm with a shell thickness of approximately 25 nm. PL spectroscopy confirmed that the synthesized composites displayed a strong eye-visible green light emission. Magnetic measurements indicated that the composite particles obtained also exhibited strong superparamagnetic behavior at room temperature. Therefore, the inner Fe_3_O_4_ core and outer Y_2_O_3_:Tb^3+^ shell layer endow the composites with both robust magnetic properties and strong eye-visible luminescent properties. These composite materials have potential use in magnetic targeting and bioseparation, simultaneously coupled with luminescent imaging.

## Background

In modern materials science, considerable attention has been paid to the precise manipulation and development of new user-friendly methods for fabricating a range of inorganic systems in the nanoscale region. Among these inorganic systems, bifunctional magnetic-luminescent composites are particularly attractive because of their unique magnetic and luminescent properties in combination in a single particle. Each bifunctional particle normally has a paramagnetic core structural domain covered by a luminescent shell domain. Unique paramagnetic properties of iron oxides have been studied intensively for many technological applications, such as jet printing, magnetic storage media, MRI contrast enhancement, hyperthermia treatment, targeted drug delivery, and cell separation [1-8]. Therefore, iron oxides (such as γ-Fe_2_O_3_ or Fe_3_O_4_) have been considered ideal candidates for core-shell structures owing to their strong paramagnetic properties. The formation of core-shell structures is followed conventionally by an encapsulation process, where the paramagnetic core is encapsulated by the silica shell layer with embedded organic dyes [9,10] or quantum dots [11,12]. On the other hand, the direct linking of a fluorescent moiety to a magnetic core normally requires the use of a sufficiently long molecular linker to bypass any possible quenching by the ferro/paramagnetic core. Furthermore, the photobleaching and quenching of organic dyes and the instability and toxicity of QDs have seriously limited the broad applications of such core-shell structures, particularly in biomedicine. Another class of a luminescent material is lanthanide-doped inorganic composites. Lanthanide-doped composites are quite promising owing to their large Stokes shift, sharp emission spectra, high luminescence quantum yield, superior photostability, and low toxicity [13,14]. Therefore, lanthanide-doped composites have become a new generation of optical probes with great potential in biomedical imaging [13].

A combination of magnetic and luminescent properties of different ceramic materials into a single composite system might enhance their application range significantly. A unique magneto-optical composite composed of a magnetite core and coated phosphor material would have great potential in both nano- and biotechnology. Up to now, there are few reports on the preparation of multifunctional composites consisting of a magnetite core with a sol–gel-coated YVO_4_:Eu^3+^ shell layer and directly linked NaYF_4_:Yb^3+^, Er^3+^ nanoparticles [14,15]. Therefore, the development of a simple and reliable synthetic method for the fabrication of bimodal nanostructures with controlled morphologies and designed chemical components is still a challenge. Moreover, magneto-optical nanostructures can provide an all-in-one diagnostic and therapeutic tool, which can be used to visualize and treat various diseases simultaneously. Another exciting application of bimodal nanocomposites is in cytometry and magnetic separation, which can be controlled and monitored easily by fluorescent microscopy.

This paper proposes a facile strategy for the fabrication of bimodal nanocomposites using Fe_3_O_4_ spheres as a core and a thin Y_2_O_3_:Tb^3+^ layer phosphor coating as the shell structure. Morphological, structural, and chemical analyses of the synthesized nanocomposites were performed using a range of microscopy and energy-dispersive X-ray analysis techniques. As the main focus of this study, the magnetic and optical properties of synthesized nanocomposites are also discussed in detail. Moreover, the simple approach presented in this paper can be applied to the fabrication of Y_2_O_3_ thin layers doped with other rare-earth ions or even for different rare-earth host oxides. Therefore, the synthesized bimodal magneto-optical system appears to be promising for magnetic separation and the diagnostic targeting and tracking of drug delivery.

## Methods

### Synthesis of core-shell Fe_3_O_4_@Y_2_O_3_:Tb^3+^ particles

All chemical reagents used in this study were of analytical grade (Sigma-Aldrich, St. Louis, MO, USA) and used as received. Spherical magnetic Fe_3_O_4_ particles were prepared using a solvothermal method according to reported protocols [15,16]. Core-shell Fe_3_O_4_@Y_2_O_3_:Tb^3+^ particles were further prepared using a facile urea-based homogeneous precipitation method [17-19]. In a typical process, rare-earth nitrates (0.0005 mol, Y/Tb = 99:1 mol%) were added to 40 ml of deionized (DI) water. Subsequently, 0.3 g of urea was dissolved in the solution with vigorous stirring to form a clear solution. The as-prepared Fe_3_O_4_ particles (50 mg) were then added to the above solution under ultrasonic oscillation for 10 min. Finally, the mixture was transferred to a 50-ml flask, sealed and heated to 90°C for 1.5 h. The resulting colloidal precipitates were centrifuged at 4,000 rpm for 30 min. The precipitates were washed three times each with ethanol and DI water and dried at 70°C for 24 h under vacuum. The dried precipitates were calcined in air at 700°C for 1 h.

### Physical characterization

The structure of the samples was examined by X-ray diffraction (XRD;D8 Discover, Bruker AXS GmbH, Karlsruhe, Germany) with Cu Kα radiation (*λ* = 0.15405 nm) and with a scan range of 20° to 60° 2*θ*. The morphology of the particles was characterized by field emission transmission electron microscopy (FETEM;JEM-2100 F, JEOL Ltd., Tokyo, Japan). The elemental properties of the samples were characterized by energy-dispersive X-ray spectroscopy (EDX;EMAX 6853-H, Horiba Ltd., Kyoto, Japan). Photoluminescence (PL;F-7000, Hitachi High-Tech, Tokyo, Japan) excitation and emission measurements were performed using a spectrophotometer equipped with a 150-W xenon lamp as the excitation source. Size measurements were performed using the Malvern Zetasizer Nano ZS machine (Malvern, UK). Magnetization measurements were performed using a quantum design vibrating sample magnetometer (QD-VSM option on a physical property measurement machine, PPMS 6000). All measurements were performed at room temperature.

## Results and discussion

### Morphology and structural properties

**Figure 1:**
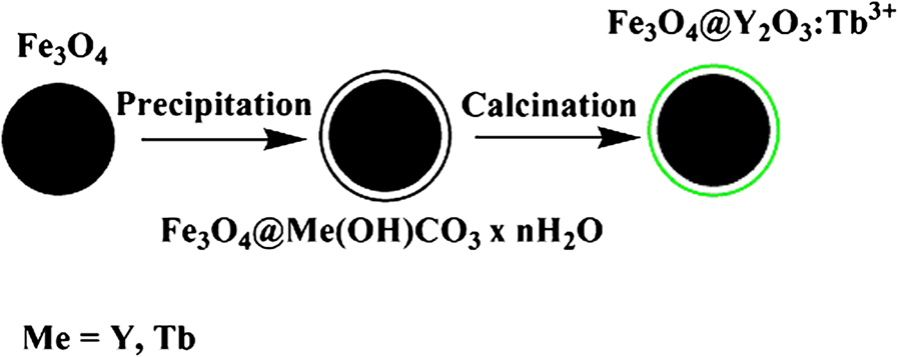
**Schematic illustration of the formation of Fe**_**3**_**O**_**4**_**@Y**_**2**_**O**_**3**_**:Tb**^**3+**^**composite particles.**

Figure 1 presents the overall synthesis procedure. First, magnetic Fe_3_O_4_ particles were prepared solvothermally as the cores. Second, a facile urea-based homogeneous precipitation method was used to form a thin uniform Y,Tb(OH)CO_3_·*n*H_2_O layer on the surface of the Fe_3_O_4_ particles. Third, bifunctional Fe_3_O_4_@Y_2_O_3_:Tb^3+^ composite particles with a core-shell structure were obtained after thermal treatment at 700°C for 1 h.

**Figure 2:**
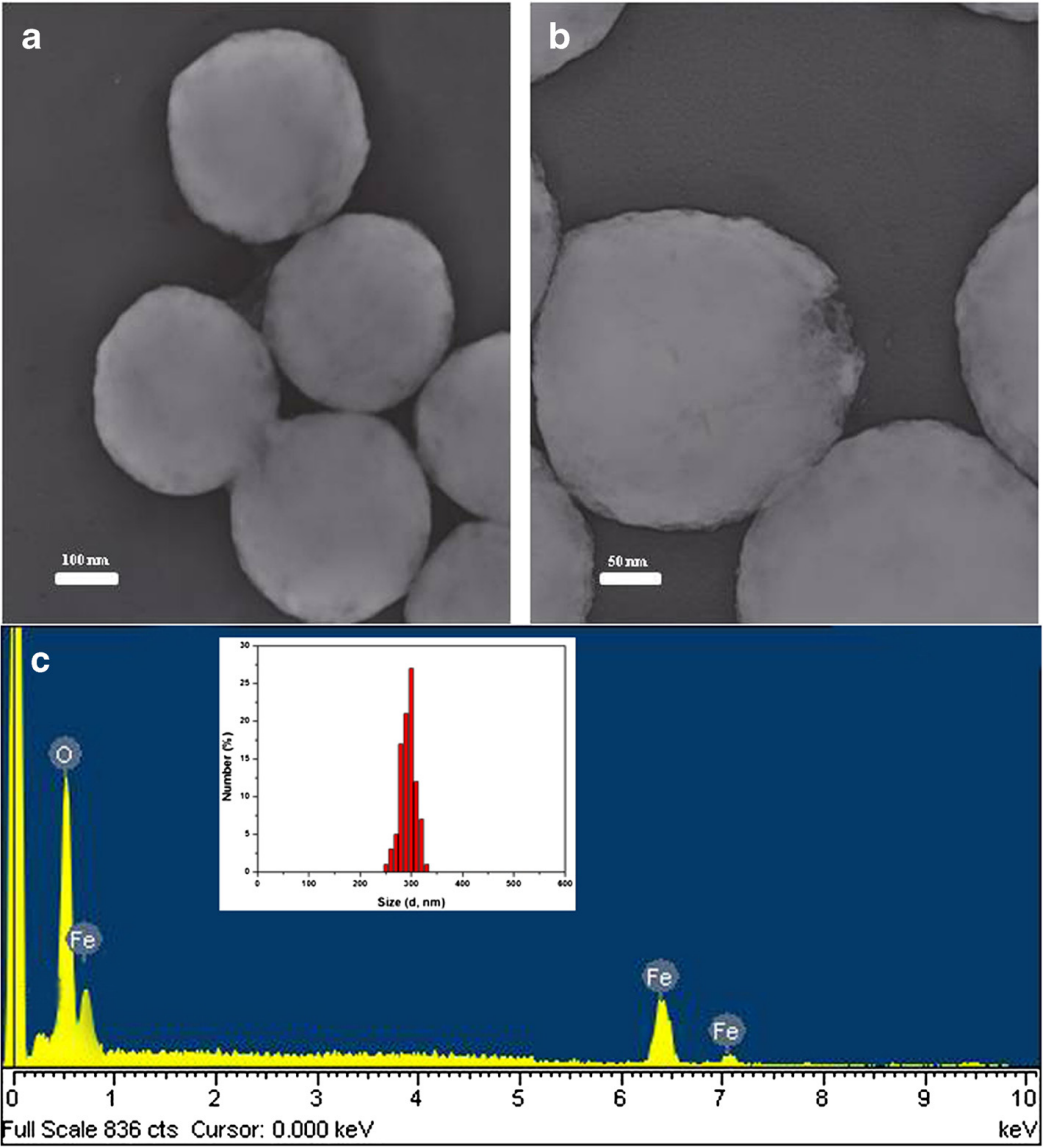
**FETEM and EDX images of Fe**_**3**_**O**_**4**_**particles. (a)** Low and **(b)** high magnifications of FETEM images and **(c)** EDX analysis and Fe_3_O_4_ size distribution (inset).

Figure 2 shows FETEM images of pure Fe_3_O_4_ microspheres with different magnifications together with the results of EDX analysis. The as-formed Fe_3_O_4_ consisted of well-separated microspheres with a mean particle size of 300 nm and a rough surface. EDX confirmed the presence of iron (Fe), oxygen (O), and carbon (C) (signal from the organic solvent).

**Figure 3:**
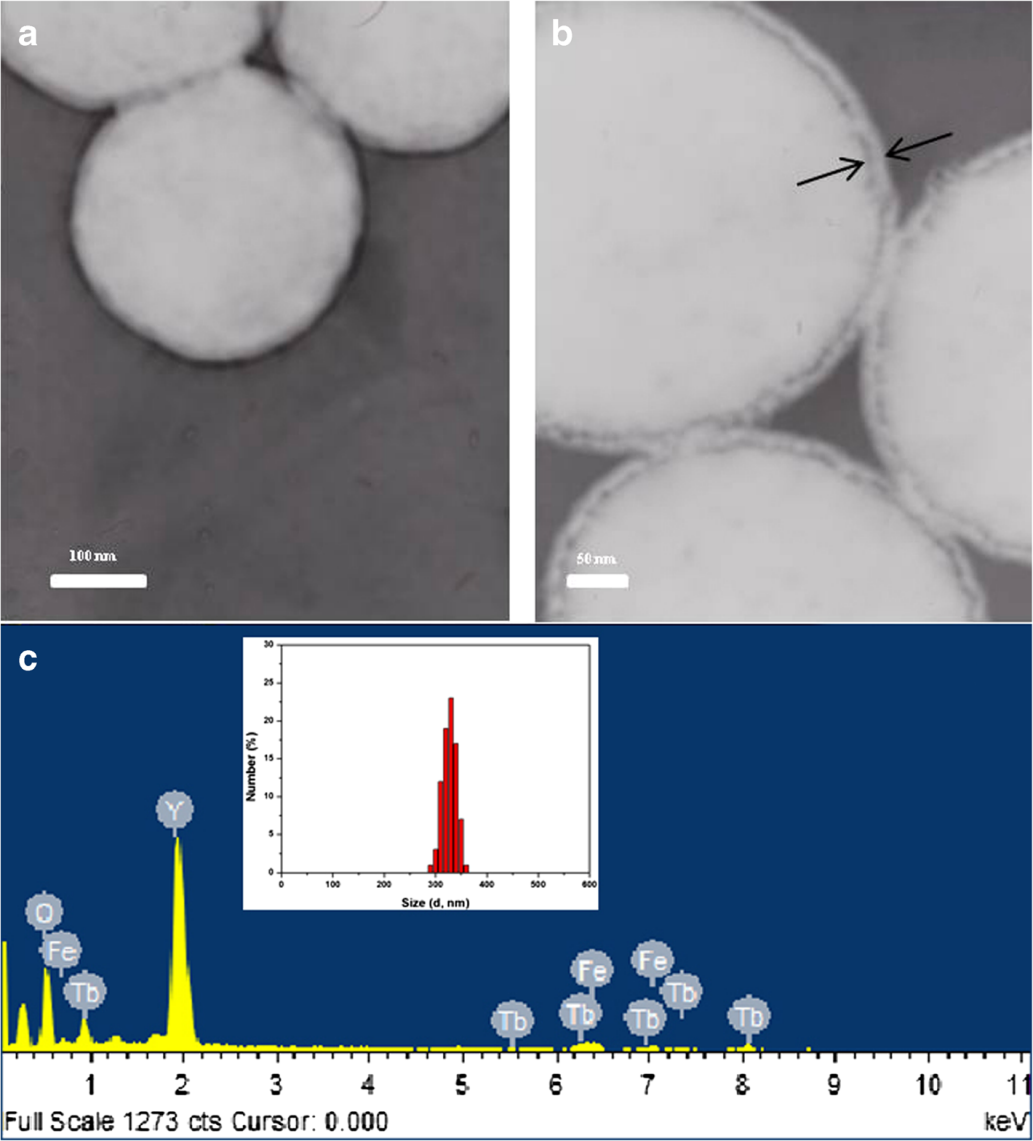
**FETEM and EDX images of Fe**_**3**_**O**_**4**_**@Y**_**2**_**O**_**3**_**:Tb**^**3+**^**particles. (a)** Low and **(b)** high magnifications of FETEM images and **(c)** EDX analysis and Fe_3_O_4_@Y_2_O_3_:Tb^3+^ size distribution (inset).

After coating with an ultrathin Y_2_O_3_:Tb^3+^ layer, the resulting core-shell Fe_3_O_4_@Y_2_O_3_:Tb^3+^ composite particles still maintained the spherical properties of the core Fe_3_O_4_ particles. On the other hand, the resulting Fe_3_O_4_@Y_2_O_3_:Tb^3+^ composite particles were slightly larger (approxi-mately 325 nm) than the bare Fe_3_O_4_ microspheres because of the additional coated layer of Y_2_O_3_:Tb^3+^, as shown in Figure 3. Moreover, the core-shell structure can also be observed clearly due to the small gap between the cores and shells. In addition, EDX analysis of the Fe_3_O_4_@Y_2_O_3_:Tb^3+^ composite particles revealed the presence of yttrium (Y), terbium (Tb), iron (Fe), and oxygen (O) in the final composite particles.

**Figure 4:**
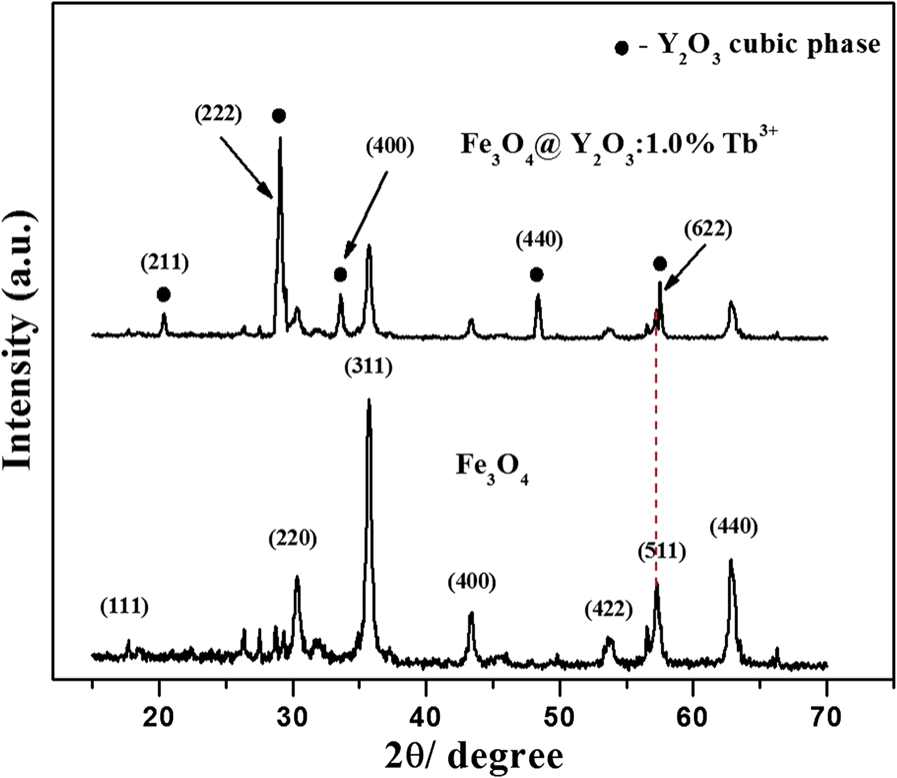
**X-ray diffraction patterns of bare Fe**_**3**_**O**_**4**_**and Fe**_**3**_**O**_**4**_**@Y**_**2**_**O**_**3**_**:Tb**^**3+**^**particles.**

XRD was used to investigate the structure and composition of the synthesized particles. Figure 4 shows XRD patterns of the bare Fe_3_O_4_ and Fe_3_O_4_@Y_2_O_3_:Tb^3+^ composite particles. The bare magnetite cores were indexed to the face-centered cubic (*Fd3m* space group) magnetite structure (JCPDS no. 19–0629) [15,16]. In the case of Fe_3_O_4_@Y_2_O_3_:Tb^3+^ composite particles, in addition to the characteristic diffraction peaks of the cubic Fe_3_O_4_ structure, there were obvious diffraction peaks indexed to the cubic phase of Y_2_O_3_ (JCPDS no. 86–1107, marked with ●), which suggests the successful crystallization of a Y_2_O_3_:Tb^3+^ thin layer on the surface of Fe_3_O_4_ particles. In addition, no additional peaks for other phases were detected, indicating that no reaction had occurred between the core and shell during the annealing process.

### Optical and magnetic properties of core-shell Fe_3_O_4_@Y_2_O_3_:Tb^3+^ particles

According to Li et al. [20] for the Y/Tb binary systems, homogeneous nucleation of Tb(OH)CO_3_ occurs in priority and then the precipitation of Y(OH)CO_3_ largely proceeds via heterogeneous nucleation on already-formed Tb(OH)CO_3_ layer. Therefore, it was assumed that Tb(OH)CO_3_ was firstly fully deposited (1 mol%) on a Fe_3_O_4_ surface and then doped into the Y_2_O_3_ structure (after the annealing process).

**Figure 5:**
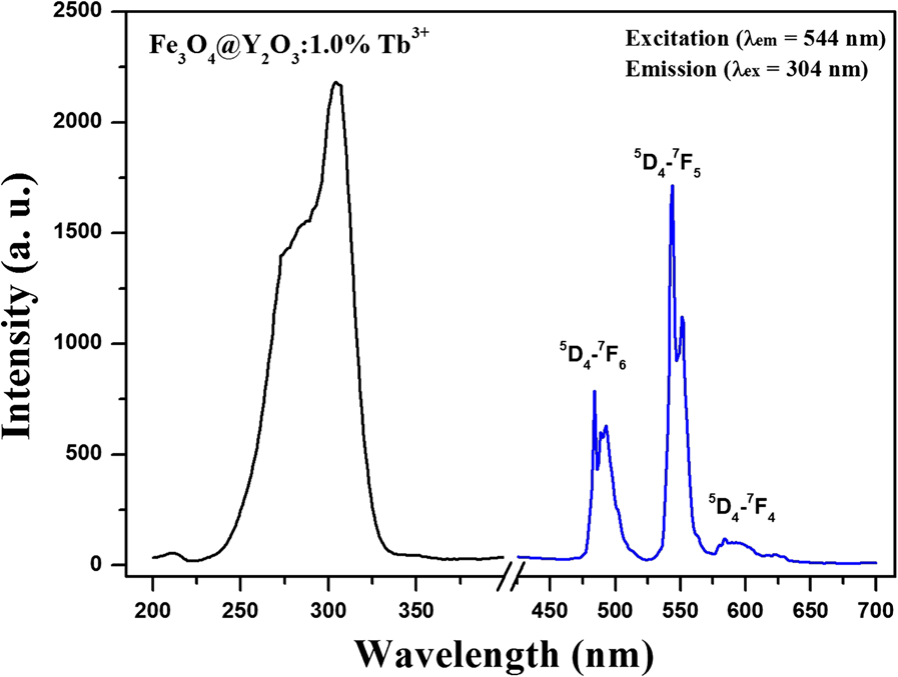
**PL excitation and emission spectra of Fe**_**3**_**O**_**4**_**@Y**_**2**_**O**_**3**_**:Tb**^**3+**^**composite particles.**

The PL properties of the core-shell Fe_3_O_4_@Y_2_O_3_:Tb^3+^ composite particles were characterized further by excitation and emission spectroscopy, as shown in Figure 5. The excitation peak of the Fe_3_O_4_@Y_2_O_3_:Tb^3+^ particles monitored at 544 nm (^5^D_4_ → ^7^F_5_ transition of Tb^3+^) consisted of a double-charge-transfer band, which was assigned to charge transfer from the 2*p* orbital of oxygen to the 4*f* orbital of Tb^3+^ between 250 and 320 nm [21,22]. The emission spectra of the Fe_3_O_4_@Y_2_O_3_:Tb^3+^ composite particles consisted of three easily distinguishable *f-f* transitions within the terbium ions. The strong green emission band with a maximum at 544 nm corresponds to the ^5^D_4_ → ^7^F_5_ transition. The blue emission at 480 to 510 nm is another characteristic of the ^5^D_4_ → ^7^F_6_ transition in Tb ions. The feeble yellow-near-red band in the range of 577 to 600 nm was assigned to the ^5^D_4_ → ^7^F_4_ transition. The characteristic emission and excitation peaks were similar to those observed in previous studies for pure Y_2_O_3_:Tb^3+^ nanocrystals, which suggest that the luminescent properties are maintained in the final composite particles [21,22].

**Figure 6:**
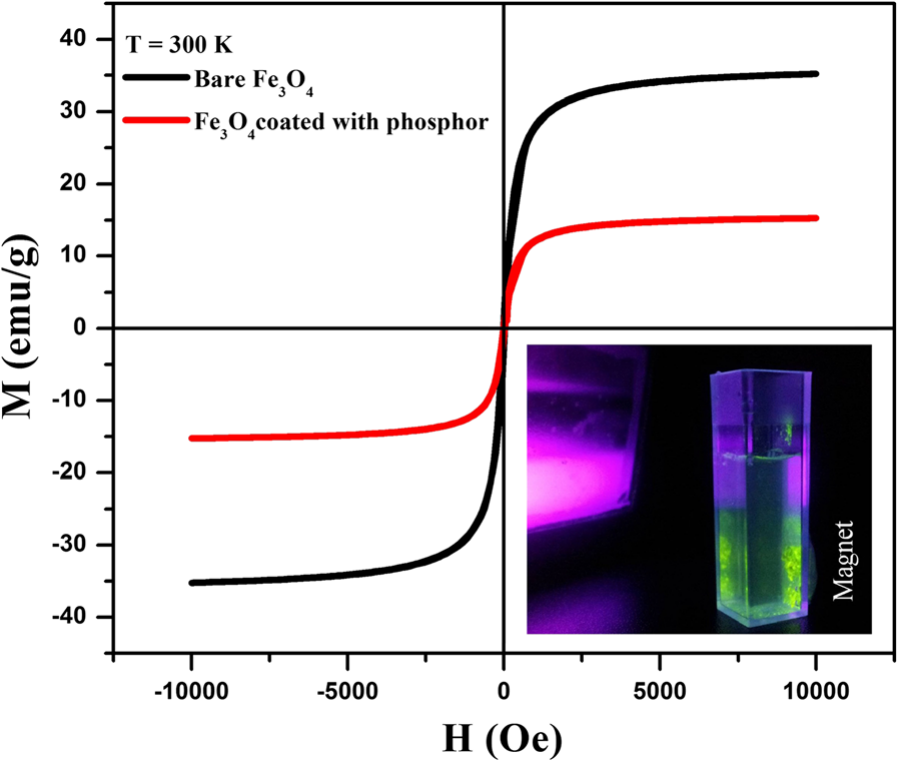
**Room temperature magnetization curves of bare Fe**_**3**_**O**_**4**_**and Fe**_**3**_**O**_**4**_**@Y**_**2**_**O**_**3**_**:Tb**^**3+**^**composite particles.**

To examine the magnetic properties of the bare Fe_3_O_4_ and core-shell Fe_3_O_4_@Y_2_O_3_:Tb^3+^ particles, the magnetization curves were measured by QD-VSM with a magnetic field cycle between −10 and +10 kOe at 300 K, as shown in Figure 6. The saturation magnetization value of the Fe_3_O_4_@Y_2_O_3_:Tb^3+^ particles was 15.12 emu/g. This value is much lower than that (34.97 emu/g) of the bare Fe_3_O_4_ due to diamagnetic Y_2_O_3_:Tb^3+^ thin shell coating. The coercivity at 300 K was negligible, indicating typical superparamagnetic behavior. Although thin shell coating reduces the magnetization of the bare Fe_3_O_4_ significantly, the Fe_3_O_4_@Y_2_O_3_:Tb^3+^ composites still showed strong magnetization, which suggests their suitability for magnetic targeting and separation. The inset in Figure 6 shows that bifunctional Fe_3_O_4_@Y_2_O_3_:Tb^3+^ composites can be attracted easily by an external magnet and show strong eye-visible green luminescence upon the excitation of a commercially available 254-nm UV lamp. Therefore, bifunctional Fe_3_O_4_@Y_2_O_3_:Tb^3+^ composites exhibit good magnetic and optical properties and have potential applications in targeting and bioseparation.

## Conclusions

Bifunctional Fe_3_O_4_@Y_2_O_3_:Tb^3+^ composites were prepared using a facile urea-based homogeneous precipitation method. These composite particles offer two distinct functionalities: an inner Fe_3_O_4_ core, which gives the composites strong magnetic properties, making them easy to manipulate magnetically, and an outer Y_2_O_3_:Tb^3+^ shell with strong luminescent properties. A similar approach can be used to develop certain bifunctional composites with different core-shell structures. In addition, the simple design concept for bifunctional composites might open up new opportunities in bioanalytical and biomedical applications.

## Competing interests

The authors declare that they have no competing interests.

## Authors' contributions

All specimens used in this study and the initial manuscript were prepared by TSA. HKK and YHH added a valuable discussion and coordinated the present study as principal investigators. All authors read and approved the final manuscript.
